# Social enrichment by separated pair housing of male C57BL/6JRj mice

**DOI:** 10.1038/s41598-020-67902-w

**Published:** 2020-07-07

**Authors:** Katharina Hohlbaum, Silke Frahm, André Rex, Rupert Palme, Christa Thöne-Reineke, Kristina Ullmann

**Affiliations:** 1 Institute of Animal Welfare, Animal Behavior, and Laboratory Animal Science, Department of Veterinary Medicine, Freie Universität Berlin, Berlin, Germany; 2 Department of Pharmacology, Charité – Universitätsmedizin, Berlin, Germany; 3 Department of Experimental Neurology, Charité - Universitätsmedizin Berlin, Berlin, Germany; 4 Unit of Physiology, Pathophysiology and Experimental Endocrinology, Department of Biomedical Sciences, University of Veterinary Medicine, Vienna, Austria; 5 Research Facilities for Experimental Medicine (FEM), Charité - Universitätsmedizin Berlin, Berlin, Germany; 6 Charité 3R, Charité - Universitätsmedizin Berlin, Berlin, Germany

**Keywords:** Animal behaviour, Animal physiology

## Abstract

Laboratory male mice are often housed individually due to aggressive behavior or experimental requirements, though social isolation can cause welfare issues. As a strategy to refine housing of male mice, we introduce the separated pair housing system. A perforated transparent wall divides the cage into two compartments and allows olfactory, acoustic, and visual communication between the two mice but prevents fighting and injuries. Long-term effects of separated pair housing on well-being and distress of adult male C57BL/6JRj mice were investigated and compared with both single- and group-housed mice. Behavioral analysis after eight weeks in three different housing systems revealed no differences in burrowing performance, social interaction, anxiety, and stress hormone concentrations. However, pair-housed mice built more complex nests compared to single-housed mice and the nest position suggested that pair-housed mice preferred the close proximity to their cage mates. Moreover, pair-housed mice showed less locomotor activity compared to group- and single-housed mice. Body weight was higher in group-housed mice. All in all, no unambiguous long-term beneficial effects of pair housing on the well-being were found. However, the findings emphasized that effects of the housing systems on behavioral, physical, and biochemical parameters must be considered in the design of animal experimental studies.

## Introduction

In nature, wild male mice live solitarily or in polygamous family groups^1^. To avoid uncontrolled breeding in laboratory animal facilities, it is a common code of practice to house laboratory mice in single-sex groups^2–4^. However, inter-individual aggression can result in stress or, at worst, pain, injuries or death^5–7^. Although there are some approaches to decrease aggressive behavior in male mice, e.g. by partial cage division^8^, they often have to be separated and housed in isolation when the aggressive behavior exceeds an unacceptable level (e.g. fights, wounds, body weight loss)^7^. Besides the prevention of aggression, further justifications for housing mice individually are veterinary needs and experimental requirements^2,5^. In comparison to group-housed mice, individually housed mice show lower variances in physiological parameters like body fat and bone mineral content^9^ and adapt faster to stress induced by cage cleaning^10^; nevertheless, single housing can also diminish well-being of mice. Social isolation is associated with depressive states^11–13^, lower tolerance to external stressors^14^, increased stress-related corticosterone concentrations^15^, and varying occurrence of anxiety-related behavior^13,16,17^ in mice.

In the scope of refinement, single housing “should be limited to the minimum period necessary, and where possible, visual, auditory, olfactory, and tactile contact with compatible conspecifics should be provided”^2^. If individually housed adult mice have the choice between an empty cage or a cage inhabited by another male, they prefer to be close the other mouse^18,19^. Therefore, one strategy to ameliorate the negative effects of single housing on the well-being of mice is to allow the animals social contact with conspecifics. A cage divider, e.g. a grid, separating the cage into two compartments prevents fighting and injuries but makes sensory contact possible. Short-term effects of this housing condition (hereafter referred to as separated pair housing) have already been investigated: heart rate, body temperature, motor activity, body weight, and nest building behavior were altered in 8–9-month-old vasectomized Hsd:NMRI mice when being housed separated by a grid with sensory contact to an unfamiliar male for 10 days^20^. Moreover, normalization of heart rate was delayed after the end of this observation period^20^. Although these effects indicated social stress and, subsequently, reduced animal well-being during the habituation to the new housing condition, long-term effects are unknown. Social stress may increase, but it is also possible that mice get used to the new cage mate living beyond the grid and benefit from its company. To investigate whether this housing condition can foster well-being of male mice, we compared long-term effects of housing adult male C57BL/6JRj mice in pairs, separated by a perforated transparent wall, which allows olfactory, acoustic, and visual communication, to single- and group-housed mice. We systematically assessed well-being of the mice using physical, biochemical, and behavioral parameters, whereby any additional stress caused by the test battery was minimized by using non-invasive methods. We analyzed burrowing and nesting, trait anxiety-related behavior in the free exploratory paradigm, the ease of handling, and social behavior in a social interaction test. Additionally, body weight and stress hormone (metabolites) concentrations in feces and hair were measured.

## Material and methods

### Ethics

All animal experimentation was approved by the Berlin State Authority (“Landesamt für Gesundheit und Soziales”, permit number: G 0251/18), conducted in accordance with the Federation of European Laboratory Animal Science Associations (FELASA) guidelines and recommendations for the care and use of laboratory animals, and registered in Animal Study Registry (10.17590/asr.0000101). Sample size calculation (primary outcome measure: effect of the housing systems on hair corticosterone) was performed using package “pwr” in R (power of 80%, standardized effect size of 0.5).

### Guidelines

The study was performed according to the guidelines of the German Animal Welfare Act and the Directive 2010/63/EU for the protection of animals used for scientific purposes. The project was planned, executed and evaluated in accordance with the PREPARE (Planning Research and Experimental Procedures on Animals: Recommendations for Excellence)^21^ and ARRIVE (Animals in Research: Reporting In Vivo Experiments)^22^ guidelines. Furthermore, the Charité Animal Welfare guidelines were followed^23^.

### Animals

A total number of 60 adult male C57BL/6JRj mice obtained from Janvier Labs (Saint-Berthevin Cedex, France) at 4 weeks of age were allowed to habituate to our animal facility for three weeks. This strain was chosen since C57BL/6JRj mice are the most commonly used laboratory mice. The mice were assigned to three study groups by simple randomization: single housing (n = 16), group housing (n = 16), and pair housing (n = 16). Twelve mice served as target animals in the social interaction test.

### Housing conditions

Mice were housed in individually ventilated cages (IVCs). The cages contained wooden bedding material (Safe^R^ Select, Safe, Augy, France),nestlets (Ancare, UK agents, Lillico, United Kingdom), and a red, triangular plastic house (length: 12,5 cm, width: 11 cm, height: 6 cm; Tecniplast, Italy) or a plastic tunnel (length: 10 cm, diameter: 4,5 cm, in-house fabrication). The animals were maintained under standard conditions (room temperature: 22 ± 2 °C; relative humidity: 55 ± 10%) on a light:dark cycle of 12:12 h of artificial light (lights on from 6:00 a.m. to 6:00 p.m.). The mice were fed pelleted mouse diet ad libitum (V1534-000, Ssniff, Soest, Germany) and had free access to tap water at all times.

**Figure 1:**
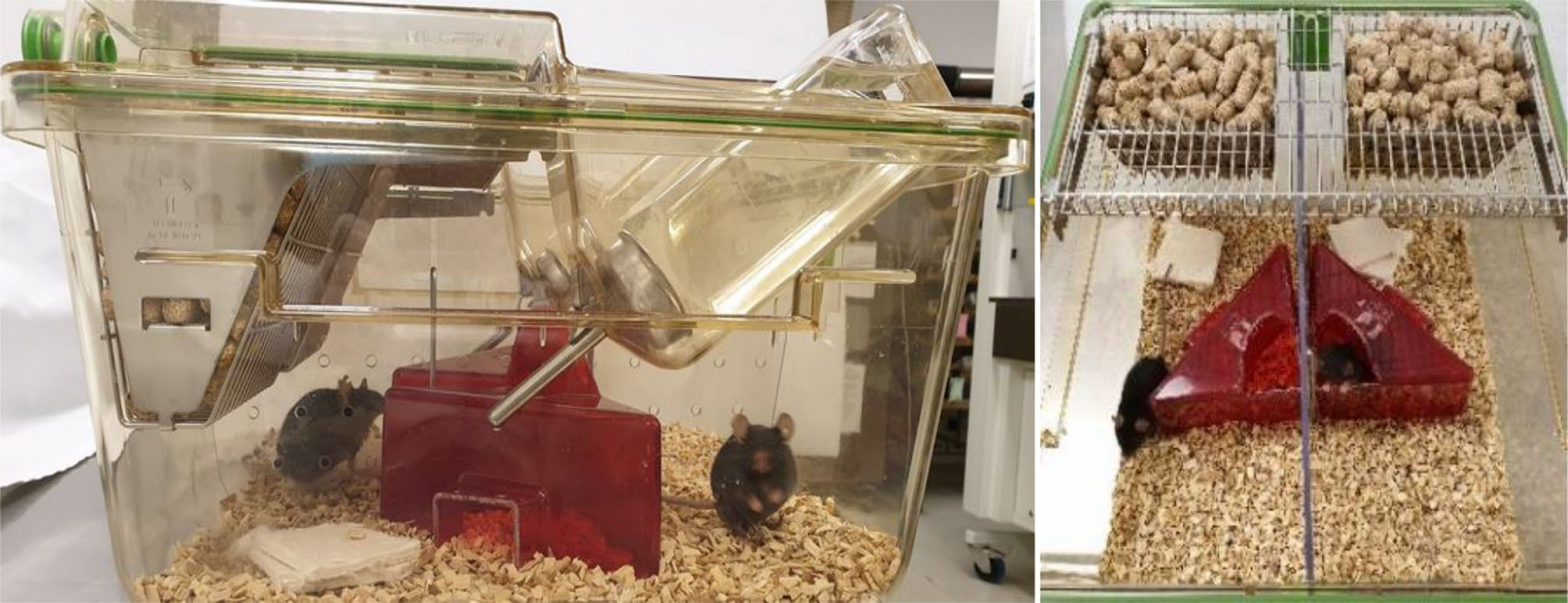
Pair housing system from the side view (**a**) and top view (**b**). Photograph: Kristina Ullmann.

During habituation, mice were kept in Polysulfone type II long cages (32 cm × 17 cm × 13 cm) in groups of 3–4 siblings per cage. After habituation, mice assigned to the study group “group housing” remained in this group during the entire period of time, i.e. their group constellation was not changed after the habituation period. Single-housed mice were transferred to Polysulfone type I long cages (32 cm × 13 cm × 13 cm) and pair-housed mice to Green Line IVC Sealsafe PLUS Rat GR 900, which were separated in two compartments (size of each compartment: 31 cm × 17 cm × 15 cm) by a perforated transparent wall (cage divider) allowing olfactory, acoustic, and visual communication (Fig. 1). Mice sharing a pair-housing cage did not have any contact to another prior to the experiment.

### Testing schedule

**Figure 2:**
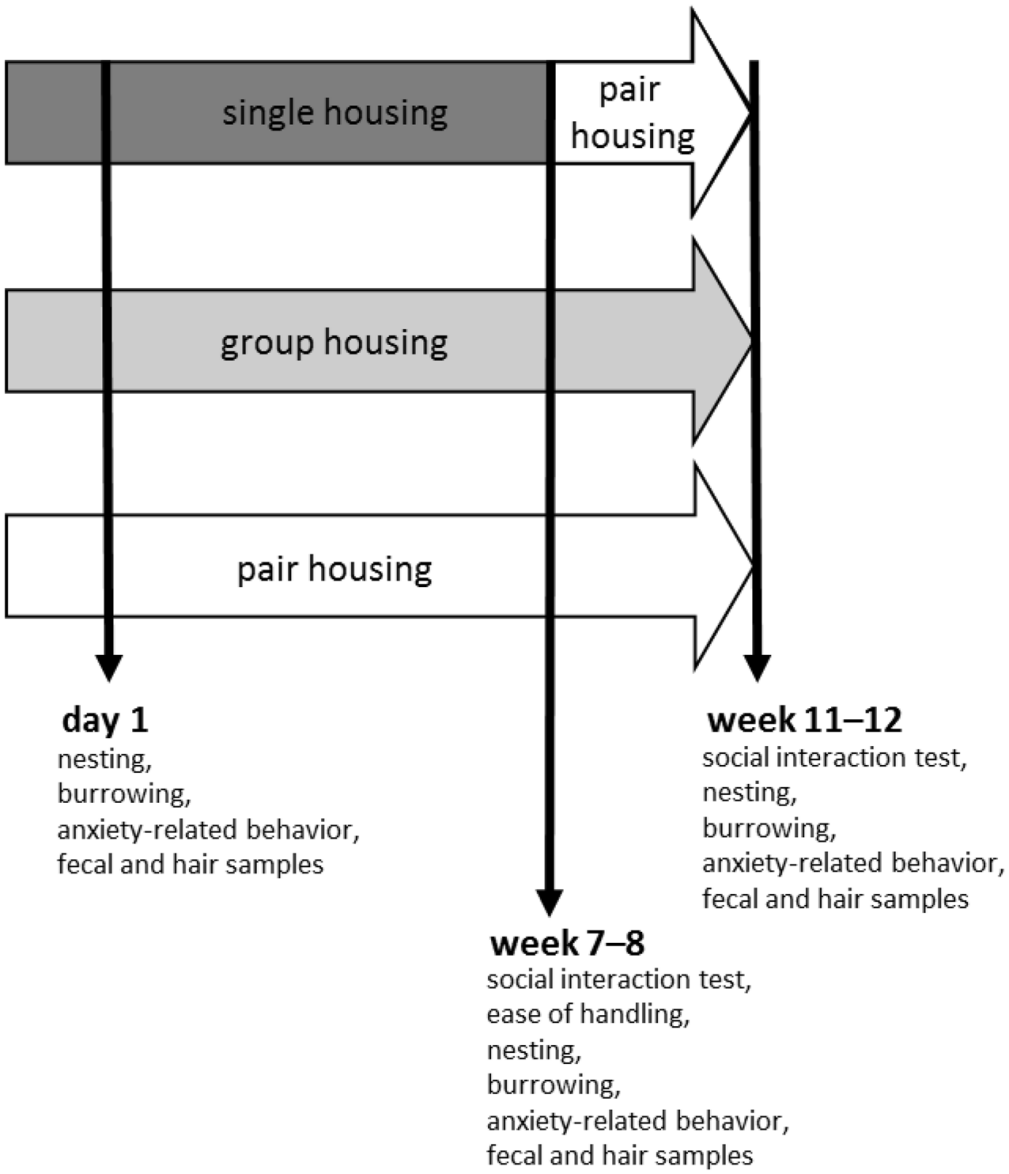
Testing schedule. Behavioral, biochemical, and physical parameters were measured to investigate the well-being of mice kept in single, group, and pair housing one day, 7–8 weeks and 11–12 weeks after transfer to the indicated housing systems.

Figure 2 gives on overview of the testing schedule. After mice had habituated to the animal facility for approximately three weeks, they were transferred to the respective housing system. The first set of analysis was performed the following day, i.e. day 1. To score nest complexity in the morning of day 1 (i.e. day 1, 7.00 and 7.30 A.M.), cages only contained nestlets but did not contain houses or tunnels during the first day in the (new) housing system. Subsequent to nest scoring, mice were moved to the testing room and were allowed to habituate for approximately an hour (~ 7.30 to 8.30 A.M.). They were then transferred to the testing cage and acclimatized for 30 min (~ 8.30 to 9 A.M.) to the new environment, prior to testing burrowing (~ 9.00 to 11.00 A.M.). After burrows were removed from the testing cages, the free exploratory paradigm for trait anxiety-related behavior was carried out. All fecal pellets, mice had excreted during the period they spent in the testing cage were collected. Furthermore, hair samples were taken. Finally, the mice were transferred to their home cages and houses or tunnels were added.

In week 7, the social interaction test was performed and a week later, in week 8, the ease of handling, nesting, burrowing, and trait anxiety-related behavior were tested and fecal as well as hair samples were taken. After week 8, single-housed mice were re-socialized and transferred to the pair-housing system (hereafter also referred to as single/pair housing). In week 11, all mice were tested again in the social interaction test and, in week 12, the other parameters, except from the ease of handling, were investigated for a third time.

Body weight was determined once a week when cages were changed. All efforts were made to blind experimenters wherever possible. Unfortunately, the study design did not allow us to blind experimenters for nesting and ease of handling tests since these investigations were carried out in the home cage of the animals.

### Burrowing

The burrowing performance^24,25^ was tested by using a blue opaque plastic water bottle as burrow (7 cm × 7 cm × 11.5 cm, 3 cm diameter of bottle neck) which was filled with approximately 140 ± 2 g food pellets normally supplied as diet^26,27^. After a 30-min habituation period to the testing cage (Polysulfone type III cages, 42 × 26 × 15 cm, approximately 0.5–1 cm bedding material, water bottle, feeder filled with a few pellets), the burrow was placed in a corner, parallel to the left long wall of the cage and the mice were allowed to burrow for two hours. At the end of this test, the weight of food pellets removed from the burrow was determined.

### Nesting

When cages were routinely changed (i.e. once a week), a square cotton nestlet of 2.4–2.6 g (Ancare, Bellmore, NY, USA) was placed in the front left corner. In the morning of the following day (i.e. approximately 1–1.5 h after lights turned on), nest complexity was scored on a 6-point-scale using the protocol by Hess et al.^28,29^. Moreover, the position of the nest was noted in pair-housed mice to evaluate whether they prefer distance or proximity to the other mouse.

### Ease of handling

The ease of handling was tested during the weekly cage change in week 8. The voluntary approach and interaction of the mice with the experimenter’s hand can provide valuable information on the “anxiety-related behavior in anticipation of handling”^30^. The cage was transferred to a table and the lid, the grid, as well as the house were removed. With the palm facing downwards, the left gloved hand was placed on the cage side opposite to the nest location. Pair-housed mice were tested simultaneously, i.e. the left hand was used for the left compartment and the right hand for the right compartment. The mice were monitored for 60 s and the latency to first voluntary interaction with the experimenter’s hand, i.e. the contact of whiskers, nose, and/or paws with the hand, was noted. Moreover, within the 60-s testing period, their interaction with the hand was scored on a rating scale for voluntary interaction:

- Score 0 = The mouse explored the experimenter’s hand by climbing on it.
- Score 1 = The mouse explored the experimenter’s hand by direct contact with the paws.
- Score 2 = The mouse explored the experimenter’s hand by direct contact with the whiskers and/or nose.
- Score 3 = The mouse carefully approached but did not touch the experimenter’s hand. Protected stretches towards the hand could be observed.
- Score 4 = The mouse moved away from the hand and settled down at the largest possible distance. It made no attempts to approach the experimenter’s hand.

After monitoring the interaction of the mouse with the hand, the animal was picked up by the tail and gently transferred to the back of the hand. The process of catching and picking up the mouse was scored on a 5-point scale (rating scale for capture, modified from Wahlsten et al.^31^):

- Score 0 = The experimenter caught the mouse at first attempt. The mouse showed minimal resistance to capture.
- Score 1 = The mouse escaped from the first capture attempt and completed less than one circuit of cage prior to capture.
- Score 2 = The mouse escaped from the first capture attempt and completed 1–2 circuits of cage prior to capture.
- Score 3 = The mouse escaped from the first capture attempt and completed more than 2 circuits of cage prior to capture. Occasionally, the mouse jumped onto the cage wall or the experimenter captured the mouse by the tail on the wall.
- Score 4 = The mouse jumped out of the cage.

### Social interaction

After mice were kept in the housing systems for 7 and 11 weeks, the social interaction test was carried out, which was performed in a 43.5 × 43.5 cm arena equipped with a perforated polycarbonate box (10 × 6.5 cm). The approach-avoidance behavior of a test mouse to an unfamiliar C57BL/6JRj male target mouse was recorded with a video tracking system. The social interaction test consisted of two 2.5-min sessions. In the first 2.5-min 'no target' session, the mouse was allowed to explore the open arena freely with an empty perforated polycarbonate box. For the second 2.5-min 'target' session, the target mouse was placed into the perforated polycarbonate box. The box allowed visual, olfactory, and acoustic interactions between the test mouse and the target mouse but prevented direct physical contact. A video tracking software (Ethovision, Noldus, Netherlands) was used to measure the distance the experimental mouse moved in the arena and the time it spent in the “interaction zone” around the target box (26.0 × 14.5 cm) in the arena. Social interaction was defined as time spent in the interaction zone during the time the target mouse was present.

### Anxiety-related behavior

Trait anxiety-related behavior was examined in the free exploratory paradigm^26,32^. The test was carried out in the testing cage after the burrow had been removed. A gridded cage lid (type I long, 34.5 cm × 14.5 cm) was placed in the testing cage and attached to the back wall using wire. The lid served as a novel object and ladder. Video cameras were installed in a distance of approximately 1.5 m. The mice were video-monitored for 5 min and the latency to explore and total duration of exploration were manually analyzed using ethological analyses software (Etholog version 2.2.5; Ottoni 1999). Experimenters were present during the test and stood silently next to the cameras.

### Analysis of fecal corticosterone and testosterone metabolites

Fecal pellets excreted during the period of approximately three hours (~ 8.30 to 11.30 A.M.), during which the mice had been individually kept in the testing cage (Makrolon type III cages, 42 × 26 × 15 cm) to investigate burrowing and perform the free exploratory paradigm, were used to analyze fecal corticosterone (FCMs) and testosterone metabolites (FTMs). After this period, testing cages were stored at room temperature for 20–24 h before fecal pellets were collected from the cages using forceps. FCMs^33–35^ and FTMs^36^ were extracted and analyzed as previously described.

### Analysis of hair corticosterone and testosterone

An electric shaver for small animals (Aesculap Isis GT 420, Suhl, Germany) was utilized to cut off hair samples (approximately 7.5 mg of hair). The first hair sample was taken from the back. Since the hair took longer than two months to fully regrow, hence there was not enough material was present at the back for the second sample in week eight. Therefore, the second hair sample was collected from the right hind leg. In week twelve of the experiment, the third hair sample could be again taken from the back, the same body location as the first sample.

Hair corticosterone and testosterone [pg/mg] were analyzed by liquid chromatography-mass spectrometry in the laboratory of Prof. Kirschbaum, Department of Psychology, Technische Universität Dresden, Germany, as described previously^37^.

### Statistical analysis

Statistical analysis was performed with IBM SPSS Version 25 (IBM Corporation, Armonk, NY, USA) and explorative data analysis and tests for normality were performed for each parameter. Differences between the groups were analyzed using the respective test, as indicated in the results section (repeated measures ANOVA, two-way or one-way ANOVA, Friedman’s two-way analysis of variance by ranks, Kruskal–Wallis-Test, Mann–Whitney-U-Test or unpaired Student t-test). Differences were considered significant at *p* < 0.05.

## Results

### Burrowing

Since the mice were tested under slightly varying conditions, i.e. the (cage position in the testing room, in the burrowing paradigm, we checked whether the cage position affected the parameters of these tests. Kruskal–Wallis-Test revealed that the cage position had no significant impact on the burrowing performance (Chi^2^ = 5.902, *df* = 5, *p* = 0.316).

**Figure 3:**
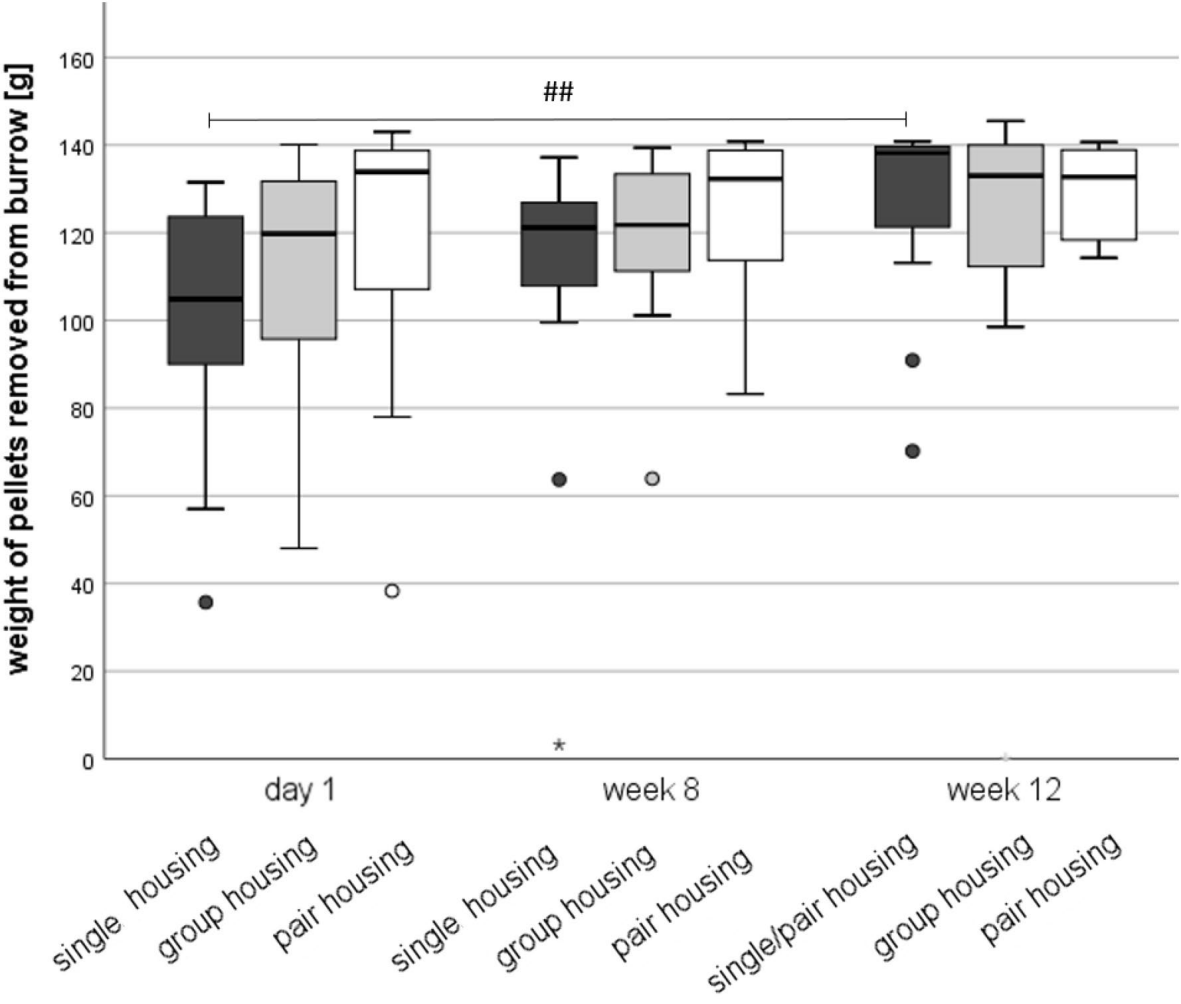
Burrowing performance. Data are presented as boxplot diagrams: the box represents the interquartile range (IQR), box edges are the 25th and 75th quartile. The whiskers represent values which are not greater than 1.5 × IQR. Dots are outliers with values between 1.5‒3.0 × IQR. Asterisks are outliers with values greater than 3.0 × IQR. Single housing: n = 16, group housing: n = 12, pair housing: n = 15. Overall, five mice were excluded from statistics because four of them removed less than 5 g food pellets from the burrow (non-responders) and one (group-housed) mouse had to be removed from the experiment due to fight-associated wounds. ^##^*p* < 0.01 versus day 1 (Friedman’s two-way analysis of variance by ranks).

Neither on day 1 (Kruskal–Wallis-Test: Chi^2^ = 5.295, *df* = 2, *p* = 0.071), in week 8 (Chi^2^ = 2.806, *df* = 2, *p* = 0.246), nor in week 12 (Chi^2^ = 0.433, *df* = 2, *p* = 0.805) after transfer to the housing systems did the burrowing performance differ between housing systems (Fig. 3). Friedman’s two-way analysis of variance by ranks indicated that burrowing performance increased over time in single-housed mice. In week 12, the re-socialized single/pair mice removed more pellets from the burrow than they did on day 1 in single housing (Chi^2^ = 11.375, *df* = 2, z = -3.359, *p* = 0.002). This effect was only found in single/pair-housed mice.

### Nesting

**Figure 4:**
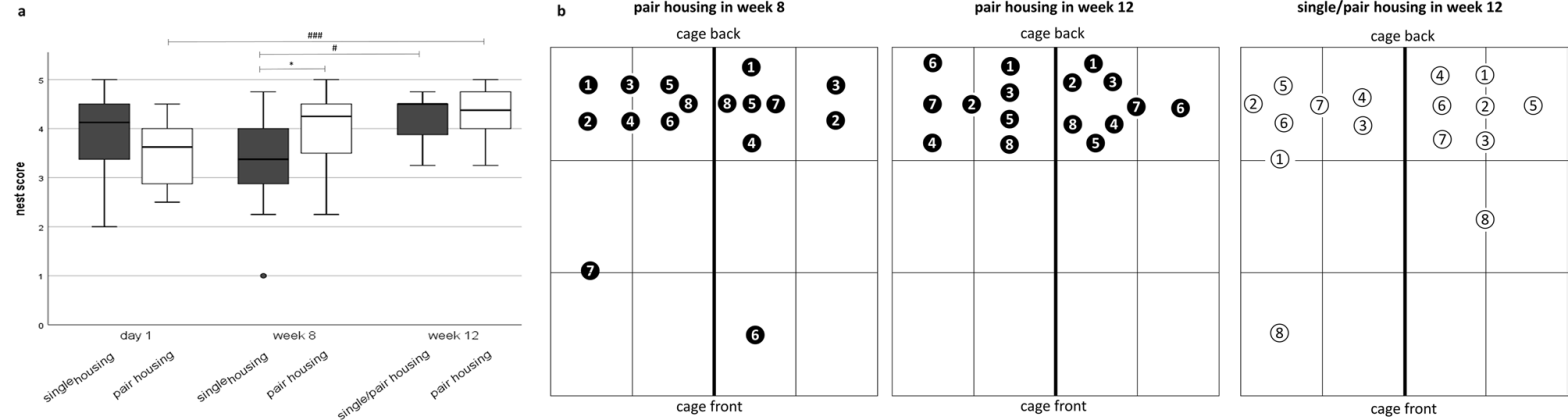
Nesting. Nest complexity scores (**a**) Data are presented as boxplot diagrams: the box represents the interquartile range (IQR), box edges are the 25th and 75th quartile. The whiskers represent values which are not greater than 1.5 × IQR. Dots are outliers with values between 1.5‒3.0 × IQR. *p < 0.05 versus pair housing (Kruskal–Wallis-Test), ^#^ *p* < 0.01, ^###^*p* < 0.001 (Friedman’s two-way analysis of variance by ranks). Single housing: n = 16, pair housing: n = 16. Nest positions in the pair housing system (**b**) both the left and right cage compartment were divided into six units. The unit (or border) where the nest was built was determined. Symbols represent a nest; the same symbols were used for nests of both mice of a pair (n = 8 pairs).

Nest complexity was only compared between single- and pair-housed mice since nests of group-housed mice were of different shape (nest scores of group-housed mice given as IQR, median; day 1: 2–4.13, 3.25; week 8: 1.75–4.00, 3.00; week 12: 2.5–3.38, 3.25). On day 1 after transfer to the housing systems, nest scores did not significantly differ between housing systems (Mann–Whitney-U = 92.500, z = –1.345, *p* = 0.184; Fig. 4a). Eight weeks after mice had been transferred to the housing systems, nests of higher complexity were found in the pair housing system (Mann–Whitney-U = 189.500, z = 2.333, *p* = 0.019). This difference was abolished when single-housed mice were transferred to pair-housing for 4 weeks (Mann–Whitney-U = 142.000, z = 0.539, *p* = 0.616). Friedman’s two-way analysis of variance by ranks revealed that nest complexity scores significantly changed over time: both re-socialized single/pair- (versus week 8, in single housing: Chi^2^ = 6.933, *df* = 2, z = -2.475, *p* = 0.040) and pair-housed (versus day 1: Chi^2^ = 18.561, *df* = 2, z = -4.066, *p* < 0.001) mice built more complex nests in week 12.

The nest positions of pair-housed mice are illustrated in Fig. 4b. Data were analyzed descriptively since mice housed as pairs had to be considered as a unit (n = 8) so that the number of units was too low for statistical analysis. Overall, the majority of mice built nests in the areas at the rear end of the cage under the food rack near the dividing wall. In week 8, two pairs built nests in units bordering one another. There were four nest pairs with a distance of ≤ 1 unit from each other and two nest pairs with a distance of 1.5–2 units. Four weeks later, four pairs continuously kept in the pair housing system built nests in units bordering one another, two pairs in units with a distance of ≤ 1 unit, and two pairs in units with a distance of 1.5–2 units. Re-socialized single/pair-housed mice showed a slightly different preference for nest location. Only one pair of nests was located next to each other. Three pairs built nests in units with a distance of ≤ 1 unit and four pairs with a distance of 1.5–2 units from each other.

### Ease of Handling

**Figure 5:**
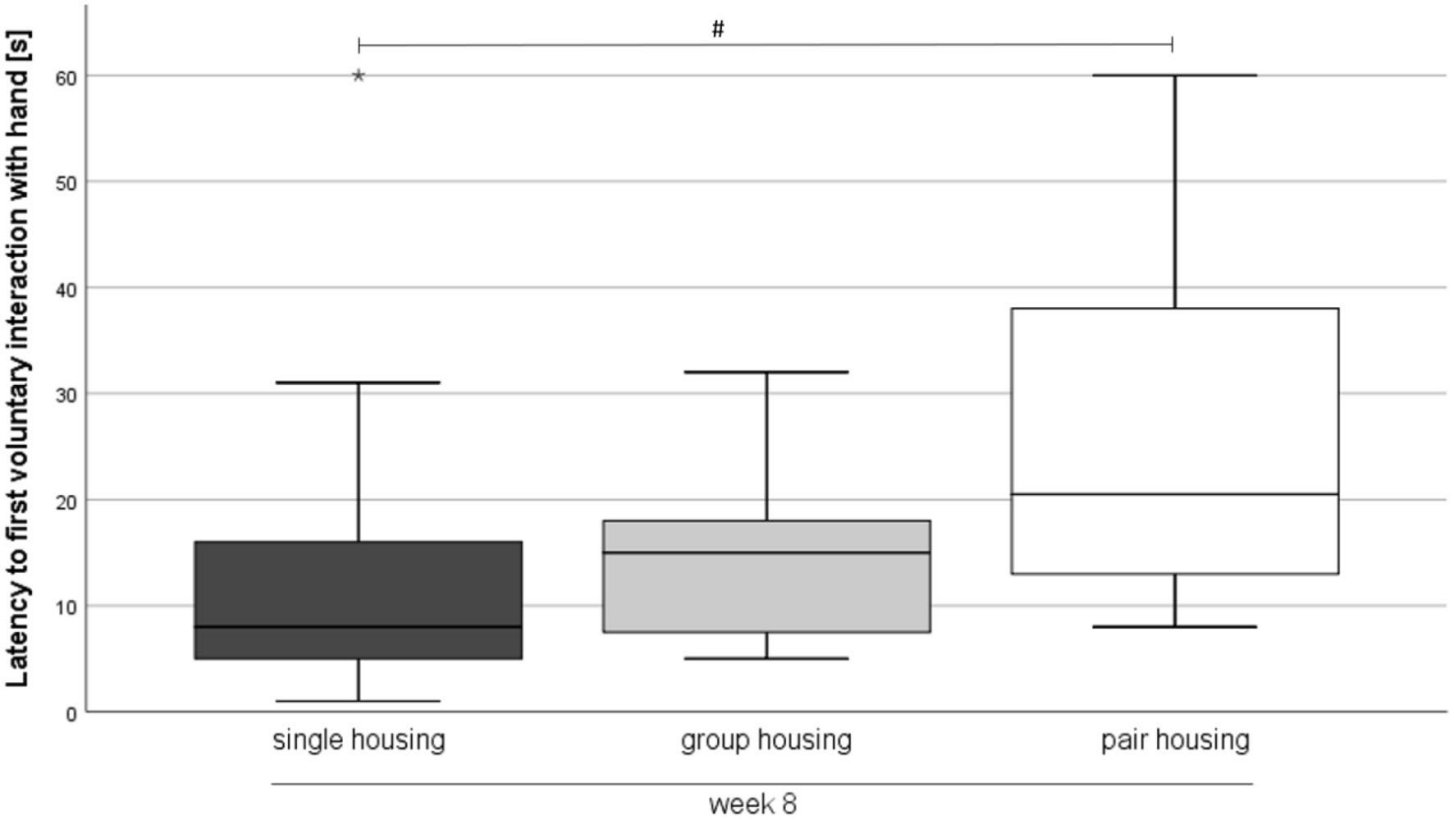
Ease of handling. Data are presented as boxplot diagrams: the box represents the interquartile range (IQR), box edges are the 25th and 75th quartile. The whiskers represent values which are not greater than 1.5 × IQR. Single housing: n = 16, group housing: n = 15, pair housing: n = 16. One mouse of the group-housed animals had to be removed from the experiment due to fight-associated wounds. # *p* < 0.05 versus pair housing (Kruskal–Wallis-Test).

Ease of handling was analyzed in week 8. While the latency to first voluntary interaction with the experimenter’s hand was significantly higher in pair-housed mice when compared to single-housed mice (Kruskal–Wallis-Test: Chi^2^ = 8.483, *df* = 2, z = -2.880, *p* = 0.012; Fig. 5), the interaction scores did not differ between single-, group, and pair-housed animals (Kruskal–Wallis-Test: Chi^2^ = 2.673, *df* = 2, *p* = 0.263). The capture score was significantly higher in single-housed mice than in group-housed mice (Kruskal–Wallis-Test: Chi^2^ = 14.340, *df* = 2, z = 3.754, *p* = 0.001; Table 1).

**Table 1:** Interaction and capture scores.

Housing condition	Interaction score^a^	Capture score^b^
Single housing	2 (2–2)	1 (1–0.25)**
Group housing	2 (2–2)	0 (0–0)
Pair housing	2 (2–2)	0.50 (0–1)

Legend: Data are given as median (25th quartile–75th quartile) and analyzed using Kruskal–Wallis-Test: **p < 0.01 versus group housing. Single housing: n = 16, group housing: n = 15, pair housing: n = 16. One mouse of the group-housed animals had to be removed from the experiment due to fight-associated wounds.

^a^Interaction score: score 0 = the mouse explored the experimenter’s hand by climbing on it; score 1 = the mouse explored the experimenter’s hand by direct contact with the paws; score 2 = the mouse explored the experimenter’s hand by direct contact with the whiskers and/or nose; score 3 = the mouse carefully approached but did not touch the experimenter’s hand; protected stretches towards the hand could be observed; score 4 = the mouse moved away from the hand and settled down at the largest possible distance; it made no attempts to approach the experimenter’s hand.

^b^Score 0 = the experimenter caught the mouse at the first attempt; the mouse showed minimal resistance to capture; score 1 = the mouse escaped from the first capture attempt and completed less than one circuit of cage prior to capture; score 2 = the mouse escaped from the first capture attempt and completed 1–2 circuits of cage prior to capture; score 3 = the mouse escaped from the capture attempt and completed more than 2 circuits of cage prior to capture; occasionally, the mouse jumped onto the cage wall or the experimenter captured the mouse by the tail on the wall; score 4 = the mouse jumped out of the cage.

### Social interaction

**Figure 6:**
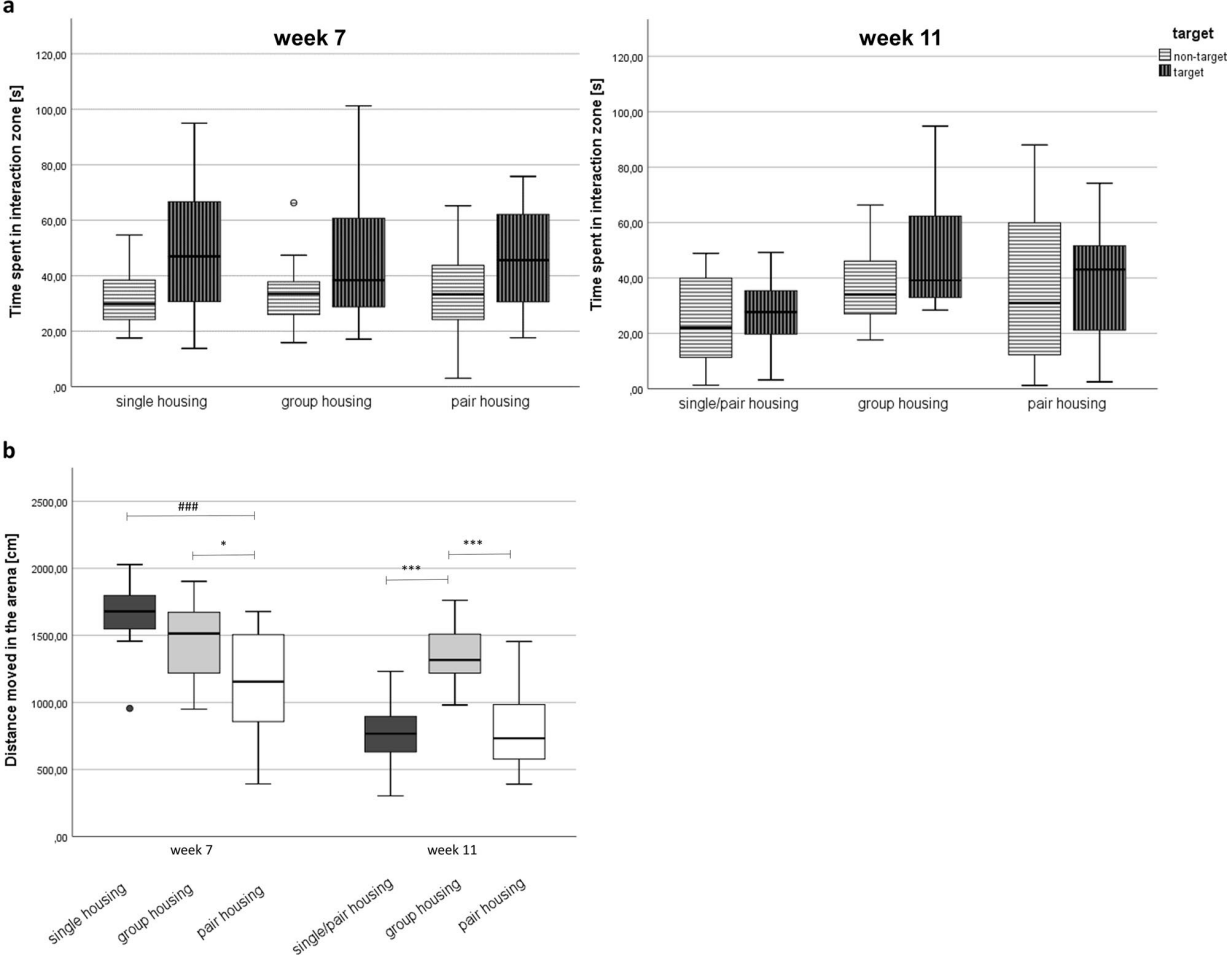
Social interaction test. (**a**) Time spent in interaction zone in week 7 (left) and week 11 (right). (**b**) Distances moved in the entire arena in absence of the target mouse in week 7 (left) and 11 (right).

All mice showed social interaction as reflected by increased time spent in the interaction zone when a target mouse was present (F(1, 90) = 9.99, *p* = 0.002; Fig. 6). However, social behavior was not affected by the housing condition (repeated measures ANOVA: F(2, 90) = 2.57, *p* = 0.82). On the contrary, locomotor activity in the absence of a target mouse differed between housing systems (F = (2, 44) = 17.21, *p* < 0.001). The distance moved in the test arena was reduced in pair-housed mice compared to single- (One-way ANOVA: F(2, 46) = 10.79, Bonferroni post-hoc analysis: *p* < 0.001) and group-housed mice (*p* = 0.025) in week 8. In week 12, both re-socialized mice (single/pair) (One-way ANOVA: F(2, 46) = 23.680, Bonferroni post-hoc analysis: *p* < 0.001) and pair-housed mice (*p* < 0.001) moved less than group-housed mice.

Data are presented as boxplot diagrams: the box represents the interquartile range (IQR), box edges are the 25th and 75th quartile. The whiskers represent values not greater than 1.5 × IQR. Dots are outliers with values between 1.5‒3.0 × IQR. *p < 0.05, ***p < 0.001 compared to group housing; ^###^*p* < 0.001 compared to single-housing (one-way ANOVA using Bonferroni post-hox analysis). Single housing: n = 16, group housing: n = 15, pair housing: n = 16. One mouse of the group-housed animals had to be removed from the experiment due to fight-associated wounds.

### Anxiety-related behavior

Since the mice were tested under slightly varying conditions, i.e. cage position in the testing room, in the free exploratory paradigm, we checked whether the cage position affected the parameters of these tests. One-way ANOVA showed that the cage position in the testing room had no significant impact on the logarithm of the latency to explore (F (5, 86) = 1.087, *p* = 0.373), the logarithm of number of explorations (F (5, 86) = 1.064, *p* = 0.386) or the total duration of exploration (F (5, 86) = 1.317, *p* = 0.265).

**Figure 7:**
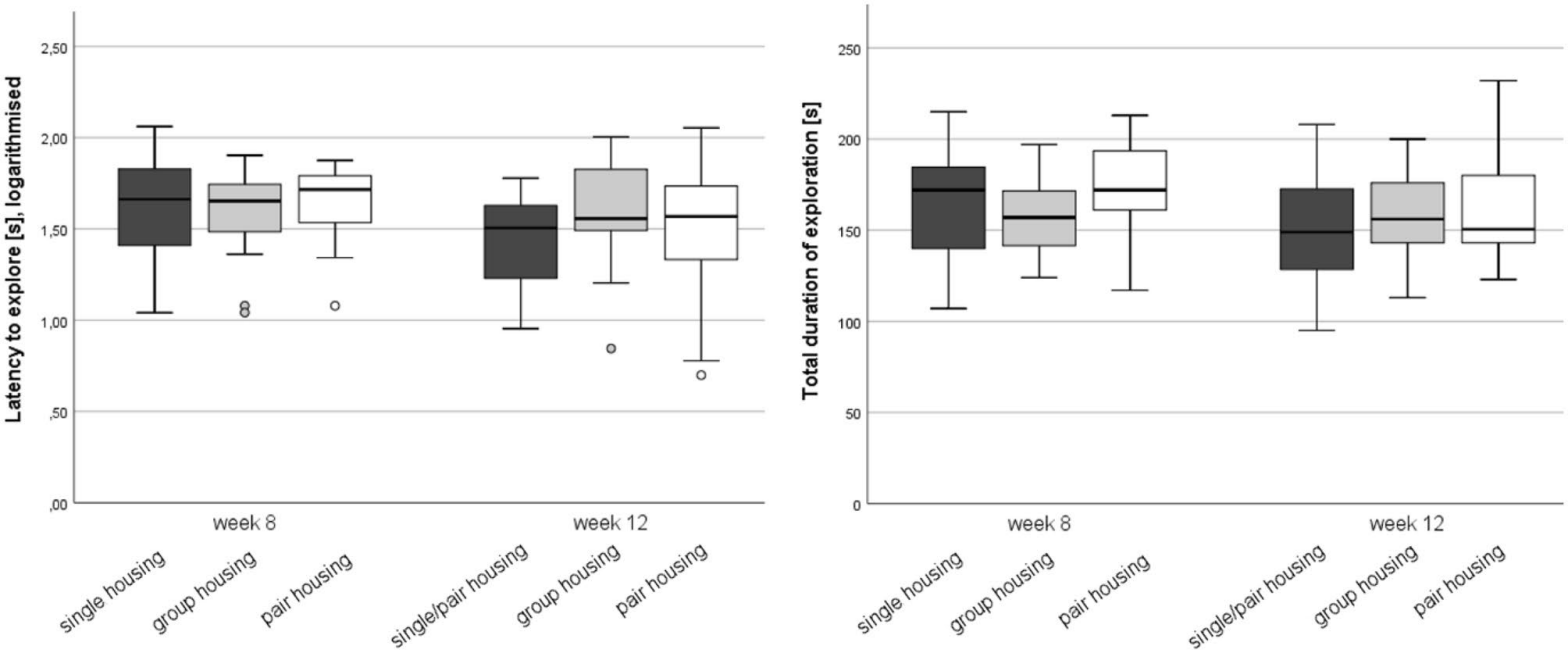
Anxiety-related behavior analyzed in the free exploratory paradigm. Latency to explore the gridded cage top (left) and total duration of exploration (right) in week 8 and 12. The right graph shows the duration of exploration Data are presented as boxplot diagrams: the box represents the interquartile range (IQR), box edges are the 25th and 75th quartile. The whiskers represent values which are not greater than 1.5 × IQR. Dots are outliers with values between 1.5‒3.0 × IQR. Single housing: n = 15, group housing: n = 15, pair housing: n = 16. One mouse of the group-housed animals had to be removed from the experiment due to fight-associated wounds; one single-housed mouse was excluded because the ladder was not properly attached to the cage wall and slipped down to the cage floor.

Repeated measures ANOVA revealed that there was no effect of time or housing system on the latency to explore (logarithmized) (time: F (1, 43) = 3.315, *p* = 0.076; housing system: F (2, 43) = 0.604, *p* = 0.551) and the duration of exploration within 5 min (time: F (1, 43) = 3.055, *p* = 0.088; housing system: F (1, 43) = 1.838, *p* = 0.171; Fig. 7).

### Body weight

**Figure 8:**
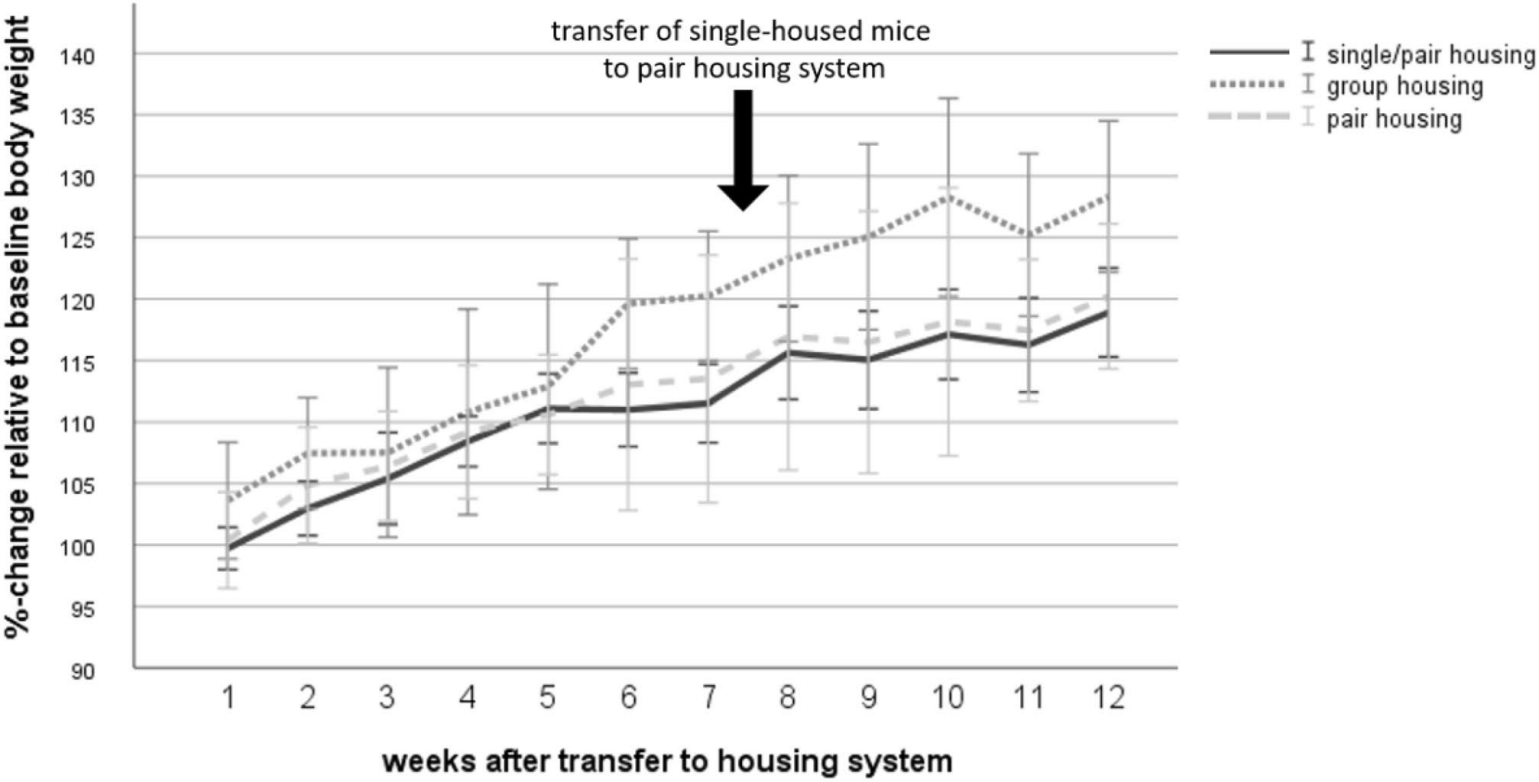
Body weight. Data are given as mean ± standard deviation. Single housing: n = 16, group housing: n = 15, pair housing: n = 16. One mouse of the group-housed animals had to be removed from the experiment due to fight-associated wounds.

During the course of the experiment, a repeated measures ANOVA with a Greenhouse–Geisser correction indicated that there was a significant effect of time on the body weight (F(1.95, 85.58) = 133.51, *p* < 0.001) and a significant interaction between time and housing system (F(3.89, 85.58) = 3.25, *p* = 0.017; Fig. 8). Tests of between-subject-effects revealed that there was a significant difference in body weight between the housing systems (F (2, 44) = 4.29, *p* = 0.020). Dunnett-T3 post-hoc analysis indicated that body weight was significantly higher in group-housed mice when compared to single-housed mice (*p* = 0.02).

The analysis of body weight change in week 9–12 also revealed a significant time effect on the body weight (repeated measures ANOVA with a Greenhouse–Geisser correction: F(1.63, 71.92) = 9.61, *p* < 0.001). Tests of between-subject-effects revealed that there was a significant difference in body weight between the housing systems (F (2, 44) = 11.98, *p* < 0.001). Bonferroni post-hoc analysis indicated that body weight was significantly higher in group-housed mice when compared to re-socialized single (single/pair) mice (*p* < 0.001) and pair-housed mice (*p* = 0.01).

### Analysis of fecal corticosterone and testosterone metabolites

A repeated measure ANOVA was conducted that examined the effect of time and housing system on FCMs or FTMs (Table 2). There was no effect of time (tests of within-subject-effects: F (2, 80) = 2.178, *p* = 0.12) and housing system (tests of between-subject-effects: F (1, 40) = 0.518, *p* = 0.60) on FCMs, whereas both time (tests of within-subject-effects: F (2, 80) = 11.655, *p* < 0.001) and housing system (tests of between-subject-effects F (1, 40) = 3.389, *p* = 0.044) significantly affected FTM levels. The post-hoc Bonferroni test revealed that FTMs concentrations were lower on day 1 when compared to week 8 (*p* = 0.003) and week 12 (*p* < 0.001). We analyzed differences in FTM concentrations between housing systems for every time point separately using one-way ANOVA with the post-hoc Bonferroni test: while FTM levels did not significantly differ between housing systems in week 8 (F (2, 40) = 0.142, *p* = 0.868) and week 12 (F (2, 40) = 2.221, *p* = 0.122), FTM concentrations were significantly lower in group-housed mice when compared to single-housed mice (F (2, 40) = 4.582, *p* = 0.016; Bonferroni post-hoc analysis: *p* = 0.048) and pair-housed mice (Bonferroni post-hoc analysis: *p* = 0.023) on day 1.

**Table 2:** Analysis of fecal corticosterone and testosterone metabolites concentrations (ng/0.05 g).

Housing system	Day 1	Week 8	Week 12	
**Fecal corticosterone metabolites**				
Single housing	45.18 ± 18.85	38.91 ± 12.70	Single/Pair housing	30.75 ± 12.84
Group housing	36.30 ± 10.05	42.49 ± 15.29	Group housing	33.29 ± 13.56
Pair housing	36.58 ± 14.87	30.98 ± 12.89	Pair housing	37.06 ± 13.98
**Fecal testosterone metabolites in ng/0.05 g**				
Single housing	3.14 ± 1.04*	3.71 ± 0.70	Single/Pair housing	3.93 ± 1.10
Group housing	2.15 ± 1.16	3.73 ± 0.59	Group housing	3.25 ± 1.22
Pair housing	3.24 ± 0.88*	3.57 ± 1.18	Pair housing	4.22 ± 1.33

Legend: Single/pair housing: After week 8, single-housed mice were re-socialized and transferred to the pair-housing system.

Data are given as mean ± standard deviation. Single housing: n = 12, group housing: n = 15, pair housing: n = 14. One mouse of the group-housed animals had to be removed from the experiment due to fight-associated wounds; four single-housed mice and two pair-housed mice were excluded because not enough sample material could be collected within the testing period.

*p < 0.05 versus group housing (one-way ANOVA).

### Analysis of hair corticosterone and testosterone

Hair samples were collected from different body regions (baseline: back, week 8: right leg, week 12: back) due to insufficient hair growth at the back. Therefore, results obtained in week 8 are not comparable with baseline and week 12 and data were analyzed for every time point separately using one-way ANOVA with the post-hoc Bonferroni test (Table 3). Hair corticosterone concentrations did not differ between the study groups at baseline when all mice were still kept in groups (one-way ANOVA: F (2, 42) = 0.498, *p* = 0.612). In week 8 (34.95 ± 5.89; F (2, 42) = 0.616, *p* = 0.545) and week 12 (F (2, 42) = 1.230, *p* = 0.303), there were no differences in hair corticosterone concentrations between single-, group-, and pair-housed mice. Repeated measures ANOVA revealed that hair corticosterone concentrations differed between baseline and week 12 (F (1, 42) = 232.97, *p* < 0.001) with higher values in week 12 (post-hoc Bonferroni analysis: *p* < 0.001).

**Table 3:** Analysis of hair corticosterone and testosterone concentrations.

Housing system	Baseline (sample location: back; pg/mg)	Week 8 (sample location: right leg; pg/mg)	Week 12 (sample location: back; pg/mg)		%-change between week 12 and baseline
**Hair corticosterone**					
Single housing	23.82 ± 3.77	37.72 ± 6.07	Single/Pair housing	34.16 ± 5.66	
Group housing	23.84 ± 4.05	36.12 ± 8.41	Group housing	29.30 ± 8.79	
Pair housing	22.63 ± 3.30	34.95 ± 5.89	Pair housing	32.88 ± 11.59	
**Hair testosterone**					
Single housing	0.66 ± 0.07*	1.28 ± 0.16	Single/Pair housing	2.07 ± 0.65**	314.47 ± 107.17^###^
Group housing	0.57 ± 0.09	1.41 ± 0.28	Group housing	2.96 ± 0.99	538.31 ± 224.81
Pair housing	0.66 ± 0.17	1.50 ± 0.50	Pair housing	2.26 ± 0.63	351.01 ± 92.12^#^

Legend: Data are given as mean ± standard deviation. Single housing: n = 16, group housing: n = 15, pair housing: n = 14. One mouse of the group-housed animals had to be removed from the experiment due to fight-associated wounds; two pair-housed mice were excluded because we could not collect enough sample material for analysis within the testing period.

*p < 0.05 (one-way ANOVA using post-hoc Dunnett T3), **p < 0.01 versus group housing (one-way ANOVA using post-hoc Bonferroni).

^#^*p* < 0.05, ^###^*p* < 0.001 versus group housing (Kruskal–Wallis-Test).

Hair testosterone concentrations significantly differed between the study groups at baseline (Welch’s F (2, 24.606) = 5.278, *p* = 0.012). Therefore, we calculated the percentage change between week 12 and baseline, which was significantly higher in group-housed mice in comparison to pair-housed mice (Kruskal–Wallis-Test: Chi^2^ = 15.637, *df* = 2; z = 2.670, *p* = 0.023) and re-socialized mice that were transferred from single to pair housing after week 8 (z = -3.860, *p* < 0.001). In week 8, no differences between the housing systems in hair testosterone concentrations were found (F (2, 42) = 1.640, *p* = 0.206).

## Discussion

Since the well-being of laboratory animals significantly contributes to the quality of research^38^, it is crucial to understand the impact of housing conditions on the well-being of mice and to further improve these. Both standard housing conditions, i.e. group and single housing, can impair well-being of male mice. Therefore, we are drawing attention to an alternative housing system as a possible refinement strategy: separated pair housing. A perforated transparent wall divides the cage into two compartments and allows for olfactory, acoustic, and visual communication between the two mice but prevents fighting and injuries. In order to enable routine use of separated pair housing in laboratory animal facilities, IVC systems were used. We systematically assessed the impact of the housing systems (group, single, pair) on the well-being of adult male C57BL/6JRj mice by investigating burrowing and nesting, trait anxiety-related behavior, the ease of handling, social behavior, body weight, and stress hormone (metabolites) concentrations in feces and hair.

The main finding of our study is the possible effect of the housing system on behavioral, biochemical, and physical parameters. In week 8, pair-housed mice built more complex nests but showed less locomotor activity and elevated anxiety-related behavior in anticipation of handling. Moreover, differences in body weight were found between single, group, and pair housing with an increased body weight gain in group-housed mice. After single-housed mice were re-socialized and transferred to pair housing, nesting and burrowing suggested that well-being of single-housed mice was improved by transferring them to pair housing.

### Transfer from group- to pair housing: Long-term effects on well-being

At 7 weeks of age, mice were assigned the study groups and hence were transferred from group housing to the respective housing systems (pair, single, group) for 8 weeks.

Since up to four mice shared a nest in the group housing system, group nests were of different structure, i.e. they were wider and flatter, than nests of individuals. Therefore, we did not compare nest complexity scores between group housing and single or pair housing but only between single and pair housing. While nest complexity did not differ between single- and pair-housed mice on day 1 after mice had been transferred to their new housing system, pair-housed mice built more complex nests in week 8. In contrast, Rettich et al. found nests of a poorer quality when 8–9-month-old vasectomized Hsd:NMRI mice were kept in pairs, separated by a grid^20^. This discrepancy may be explained by differences in age^39^ or experimental design, which deviated from Rettich et al.^20^. First, the habituation period to the housing system was much shorter in Rettich et al.^20^ than in our study, i.e. 9 days versus 8 weeks, respectively. Secondly, in contrast to our study, Rettich et al.’s mice had already experienced previous experimental phases, i.e. three 14-day periods of single-housing followed by rehousing with a female for several weeks, before they were transferred to the pair housing system with an unfamiliar male. This may have enhanced territorial authority as well as aggressive behavior^20^ causing higher distress levels than in mice of our study. Thirdly, differences in cage ventilation must be considered because the movement of air causes heat loss through convection and, therefore, can affect nest building behavior^40,41^. High nest walls can protect mice from the draft caused by high ventilation rates^41^. With regard to our study, the ventilation rate differed between IVCs used for pair housing (75 changes per hour) and IVCs used for single housing (50–60 changes per hour), which could explain the higher nest complexity found in pair-housed mice. Another important issue is that the experimenter assessing the nests could not be blinded because the pair housing system with its cage divider looks different from the cages used for single housing, even on images or videos.

To investigate whether mice could meet their need for proximity in the pair housing system, we determined the positions of the nest sites. Mice obviously preferred to build nests in the rear third of the cage under the food unit, probably due to lower light intensity (16 lx in average) in this part of the cage compared the front side (40 lx). Moreover, the draft may be less at the rear end of the cage under the air inlet, as dead-air spaces are created^41^. We found that the majority of pairs located their nests at a distance of ≤ 1 unit from each other and, interestingly, most nests were positioned next to the cage divider, which led us to the hypothesis of the mice preferring the proximity to the other male rather than staying alone. However, this may also be due to the intention of sharing body heat. It was shown earlier that male mice choose dwelling next to a familiar cage mate^19^ and nests of pair-housed male mice were located close to each other^20^.

Mice are highly social animals and usually interact with unfamiliar mice intruding into their territories. Social interaction of mice appeared not to be affected by the housing systems, as demonstrated in the social interaction test using a male mouse as novel intruder, though results are difficult to interpret as the distance moved in the entire arena in absence of an intruder differed between housing conditions. Mice kept as pairs moved less than single- and group-housed animals, which may indicate a decrease in exploratory behavior and is the reason for which the time spent in the interaction zone should be interpreted with caution. A higher locomotor activity in socially isolated C57BL/6 mice compared to group housing was previously found in behavioral paradigms such as the open field^16,17^; pair housing seemed to have the opposite effect although the findings from the social interaction test cannot be interpreted in the same way as in the open field test. Locomotor activity can be affected by stress^42–46^, and, hence, may indicate higher stress levels in single-housed mice. We did not investigate whether these effects are also present in the home cage. Therefore, home cage activity and time spent near the dividing wall should be further investigated. Moreover, it would be of high interest to monitor whether and how pair-housed mice actually interact with the other mouse beyond the dividing wall and whether the dividing wall is marked with urine more intensively than other areas of the cage to indicate territorial boundaries.

Considering the impact of the housing systems on locomotor activity, results obtained from the free exploratory paradigm also need to be carefully interpreted. Locomotor activity could not be determined in this test. In the free exploratory paradigm, all mice, independently of the housing system, explored the gridded cage lid and no effects of the housing systems on trait anxiety-related behavior was detected. In contrast, a decrease in trait anxiety was found in 12-week old male Swiss CD-1 mice that had been socially isolated for 3 weeks when compared to group-housed animals^14^. Moreover, changes in state anxiety-related behavior induced by social isolation, depending on mouse strain, age, and duration of isolation, were reported^16,47^.

To investigate whether the housing systems influenced the human-animal interaction and the ease of handling during routine husbandry procedures, we measured the latency to first voluntary interaction with the experimenter´s hand and used an interaction as well as a capture scoring system. While all mice, independently of the housing systems, explored the experimenter’s hand by direct contact of whiskers and/or nose, pair-housed mice showed a prolonged latency to first voluntary interaction when compared to single-housed mice. This may be explained by their lower locomotor activity we found in the social interaction test. Hurst and West^30^ previously used the latency to first voluntary interaction with the experimenter’s hand to assess the impact of tail, tunnel, and cup handling on the “anxiety-related behavior in anticipation of handling”. All mice used in the present study were tail handled. There was one striking difference between the cages used for the different housing systems: for handling the mice, the food unit had to be removed from the cages used for single and group housing (i.e. Polysulfone type I and II long), whereas the food unit of the Green Line IVC Sealsafe PLUS Rat cages, our pair housing system, remained in the same position. When pair-housed mice had to be removed from the cages for routine husbandry procedures, they hid under the food unit and clung tightly to the grid, as reported by our animal care technicians. One can imagine that more strength must be expended when picking up a mouse clinging to a grid than a mouse sitting on the cage floor, which may be why pair-housed mice had worse handling experiences than single- and group-housed animals and showed higher anxiety-related behavior in anticipation of handling. The potential impact of the food unit providing foothold for the mice was an incidental finding and was only revealed due to the good communication between technicians and scientists. Since it was not considered in the experimental design of the study, further investigations are needed to substantiate our interpretation of these results. The capture scores did not reflect the observations of our technicians, because the food unit was removed for this test to create equal conditions for all experimental groups. Single-housed mice were more difficult to catch than group-housed mice. Single-housed mice may be less used to moving objects in their environment because they do not share their cages with other animals. The moving hand of the experimenter may trigger their flight response to a higher extent than in pair- and group-housed mice.

FCM and hair corticosterone concentrations were used as markers for stress. Since the excretion of FCMs depends on the circadian rhythm^34^, fecal samples were always collected at the same time of day (~ 8.30–11.30 A.M.). The results reflected the hypothalamic–pituitary–adrenal axis activity within the timeframe of about 8–10 h prior to sample collection^34^, i.e. in the dark period on day 1 or week 8 after transfer to the housing systems. Hair corticosterone may not only indicate ongoing stress^48^ the mice experienced at the time when the samples were taken but also serve as a retrospective biomarker for stress, as corticosterone can accumulate in hair over time^49^. The analysis of FCMs and hair corticosterone concentrations did not reveal any significant short- and long-term effects of the housing systems on the stress hormone (metabolites) levels. Hair corticosterone increased over time, as expected^49^. Pair housing appeared not to be less stressful than single or group housing, as living next to a male rival may be as stressful as social isolation or being part of a hierarchy^50^. This is in line with other studies, which neither found changes in FCMs in male C57BL/6 J mice^51^ nor in plasma corticosterone concentrations in males CD-1^14^, male TO albino mice^52^ or male Swiss albino mice^53^ when the animals were separated. In contrast, a transient increase in urine corticosterone concentrations was found a day after male C57BL/6NCRl mice were transferred to individual cages, however, their urine corticosterone concentrations were lower in comparison to group‐housed mice on day 7^10^. When analyzing hormone (metabolites) levels, the social context and social rank of the individuals should also be considered, in order to investigate the correlation between the hormone (metabolite) levels and the social behavior of the mice^54^. Plasma corticosterone levels were demonstrated to be lower in alpha male CD-1 mice than in subordinates in despotic groups^54^. Stress levels of the latter were shown to be higher in larger groups than in groups of two mice only^54^. Against this background, it would be of high interest to further investigate whether mice establish a hierarchy in the pair housing system and whether the lack of a clear hierarchy, which is probably associated with ongoing social defeat through the cage divider, may be a reason for stress levels comparable with single and group housing.

Besides corticosterone (metabolites), testosterone is also dependent on the group structures and the individual social ranks^54^. Testosterone levels of alpha male mice in despotic groups are higher than in subordinates. A flat hierarchy and also a victory result in higher testosterone concentrations of subordinate male mice when compared to despotic group behavior and defeat^54,55^. In the initial stage of acute stress, testosterone levels can be elevated; a variety of factors, e.g. the absence of chronic stress and/or dominant status, contribute to this transient increase^55^. On day 1, FTM levels were elevated in single- and pair-housed mice when compared to group-housed animals, suggesting mild acute stress due to acclimatization to the new housing condition and, for pair-housed mice, to the unfamiliar cage mate. In latter case, the increase in testosterone levels may be due to the agonist encounter with the other male mouse. As group-housed mice were siblings, it is likely that the hierarchy in the group was flat and rank fights were rare. In contrast to single- and pair-housed mice, group-housed animals remained in their familiar group and housing condition which is why we did not expect their hormone (metabolites) levels to change on day 1. However, since we did not measure baseline FTM, it remains unclear whether the difference in testosterone levels was due to a decrease in group-housed animals or an increase in single- and pair-housed animals. Hair testosterone concentrations differed between the three study groups at baseline, so it is possible that testosterone values had already been higher in mice that were assigned to the single and pair housing group before they were transferred to the housing systems. Therefore, both hair as well as fecal testosterone values should be interpreted with caution. Since hair samples were taken from different body parts, i.e. from the back at baseline and from the right hind leg in week 8, it is not clear to the authors if values can be compared between these two time points. Hair corticosterone concentrations may depend on the sample location.

Body weight was significantly lower in single- and pair-housed mice compared to group-housed mice. A difference in body weight between single and group housing had already been demonstrated in male C57BL/6J^9,16^ and male C57BL/6JRj^17^. Along with this, it would be interesting to investigate whether the weight of single- and pair-housed mice decreased due to reduced food intake, higher activity in the home cage or enhanced thermoregulation. With regard to latter, single- and pair-housed mice may need more energy to maintain body temperature because they cannot huddle together to keep warm while resting.

Overall, we could not identify clear long-term beneficial effects of pair housing on the well-being of male C57BL6/JRj mice.

### Transfer from single to pair housing

At the age of 15 weeks, single-housed mice were transferred to the pair-housing system in order to re-socialize them, which are hereafter also referred to as single/pair housing. The effects of the transfer on their well-being were investigated after three to four weeks, i.e. in week 11 and 12 of the study. These results may be of interest for studies that require to keep mice individually for a prolonged period but would allow for re-socialization afterwards. However, re-socialization of male mice in groups usually is not possible. Therefore, the question raises whether it would be beneficial for the mice to be transferred to a pair housing system than being kept socially isolated for the rest of their lives.

Body weight remained higher in group-housed mice and no changes in trait anxiety-related behavior, and hormone (metabolite) concentrations, except for hair testosterone, were found. Hair testosterone concentrations increased in all study groups, but the percentage change to baseline was lower in (re-socialized) pair-housed mice in comparison to group-housed animals. These findings suggested that higher levels of testosterone were incorporated into the hair shaft in group-housed mice over the experimental period, which may be explained by dominance hierarchy and fights resulting in high testosterone levels in dominant mice and the subordinate animals most attacked^56^. However, concentrations of FTMs did not reflect this hypothesis. The discrepancies may be explained by the low number of sampling time points, which did not allow to determine the excretion of FTMs over the entire experimental period but only at certain time points. In contrast, the level of hair testosterone measured at a certain time can reflect several weeks of hormone accumulation^49^, which may provide more information on the hormonal balance of these mice in this case.

Nest complexity and burrowing performance suggested that the well-being of the re-socialized mice increased under the pair-housing conditions. While nest complexity was poorer in single- than pair-housed mice in week 8, there were no differences in week 12 anymore, as nest complexity significantly increased over time. This may indicate fostered well-being of single/pair-housed mice, but can also be explained by the different ventilation rates^40,41^. In contrast to pair-housed mice, the majority of re-socialized mice appeared not to prefer a nest location next to the cage divider. Rettich et al.^20^ explained the short-term negative effects of male:male pair housing on 8–9-month-old vasectomized Hsd:NMRI mice by stronger territorial authority, which may also influence their choice of nest locations. However, based on our data, we cannot conclude with certainty that the nest locations represent an indicator of stronger territorial authority due to the 8-week period of single housing. Further investigations are needed to prove this hypothesis.

Although burrowing performance did not differ between single/pair, group, and pair housing in week 12, re-socialized mice removed more pellets from the burrow when compared to day 1. Whether this is due to habituation to the experimental set-up, i.e. set-up and unfamiliar water bottle, or due to improved well-being is unclear, since FCM concentrations did not reveal any time-dependent changes in stress levels.

In week 11, mice were tested again in the social interaction test. Interestingly, re-socialization of single-housed mice reduced the locomotor activity in the entire arena in absence of an intruder when compared to group-housed mice and reached a level comparable to mice that had been kept in the pair housing systems since day 1 of the study. These findings confirmed the results we found in week 8. Moreover, however, it remains unclear whether anxiety-related behavior in anticipation of handling increased and capture scores decreased in re-socialized mice since, unfortunately, the ease of handling was not tested again in week 12 again.

In the light of previous findings of mice being capable of recovering from effects induced by single housing^57^, re-socialization seemed to improve well-being of single-housed mice to a slight extent.

## Conclusion

The results of the present study did not reveal unambiguous long-term beneficial effects of separated pair housing on the well-being of adult male C57BL/6JRj mice, though the transfer from single to pair housing appeared to slightly foster well-being with regard to nest complexity and burrowing. Taking into account that male mice prefer dwelling near other males to staying alone, pair housing rather than single housing can meet this need, as reflected by the nest locations. It should be noted that the effects of the housing systems on behavioral, physical, and biochemical parameters needs to be considered in the design of animal experimental studies and their analysis.
